# An analysis of social dimensions of podoconiosis and leprosy on affected households in endemic health districts of the North West Region of Cameroon

**DOI:** 10.1016/j.ssmph.2022.101187

**Published:** 2022-08-06

**Authors:** Ayok M. Tembei, Jonas A. Kengne-Ouafo, Bonekeh John, Theobald M. Nji, Peter Enyong, Theresa Nkuo-Akenji, Gail Davey, Samuel Wanji

**Affiliations:** a Epidemiology and Control of Infectious Diseases, Department of Microbiology and Parasitology, University of Buea, PO Box 63, Buea, Cameroon; b Research Foundation in Tropical Diseases and Environment, PO Box 474, Buea, Cameroon; c Department of Sociology and Anthropology, University of Buea, PO Box 63, Buea, Cameroon; d Wellcome Trust Centre for Global Health Research, Brighton & Sussex Medical School, Falmer Campus, Brighton, BN1 9PX, UK; e Mbebah Vigilantic Farming and Development Association in Ndop, Ngokutunja Sub-division of the North West Region, Cameroon

**Keywords:** LEPROSY, Podoconiosis, QUALITY OF LIFE, STIGMA

## Abstract

**Background:**

Podoconiosis and leprosy are Neglected Tropical Diseases associated with low quality of life, social stigma and isolation of affected people and families. Despite the substantial social burden it imposes, podoconiosis has largely been ignored in the global health literature until recently unlike leprosy. This study assessed and compared the quality of life and social impact of podoconiosis with that of leprosy among affected households and neighborhoods in North West Cameroon.

**Methods:**

A comparative cross-sectional design was used. Eighty-six households: 43 podoconiosis and 43 leprosy, plus household neighbours were enrolled from July and August 2015 from three health districts. Podoconiosis patients living in households within Batibo and Ndop health districts were sequentially sampled using a list of confirmed podoconioisis cases from previous studies. Leprosy patients living within communities in Mejang Health Area were sequentially sampled using the Mbingo treatment center register. WHO BREF tool was used to assess quality of life. Franklin Stigma Scale was adapted to assess felt and enacted stigma. Mann-Whitney U test was used to compare differences in stigma and QoL.

**Results:**

Physical domain showed a significant difference in the distribution in quality of life between groups (p < 0.05, median:70; U:635, r = 0.2). Overall enacted stigma revealed significant differences with p < 0.05 and r = 0.4. Overall stigma from family members (median:17, U:627 and r = 0.3) and neighbours (median:67, U:336 and r = 0.5) showed significant differences with p < 0.05 in the distribution of scores for both diseases. Sex and age showed significant associations with QoL and stigma.

**Conclusion:**

This study reveals the quality of life and stigma associated with podoconiosis on affected households to be comparable to that experienced by households with a leprosy patient. There is need for intensified preventive, management and control schemes to fight podoconiosis in Cameroon, just like leprosy.

## Highlights

• Podoconiosis and leprosy are NTDs linked with low quality of life, stigma and isolation of affected people and families.
• The social consequences include difficulties in finding employment, gaining education and getting married.
• Podoconiosis impairs similar social burden on its victims like leprosy, yet remains neglected in Cameroon unlike leprosy.
• This disparity in attention may be due to lack of evidence on the social impact of Podoconiosis likened to leprosy.
• This paper creates scientific basis on the social effects of Podoconiosis in Cameroon to inform health policy actions.

## 1 Introduction

Podoconiosis (endemic non-filarial elephantiasis) is a non-infectious geochemical disease caused by the conjunction of environmental, genetic, and economic factors (Davey et al., 2007). This condition has been categorized as an environmental geochemical disease resulting from irritant soil, and occurs in individuals who have been exposed to red clay soil derived from volcanic rock (Deribe et al., 2015; Price, 1990; Wanji et al., 2008). A recent systematic review described podoconiosis to exist or be endemic in 32 countries, 18 of which are from the African Region, 3 from Asia and 11 from Latin America (Deribe et al., 2018a). From the same review, overall podoconiosis prevalence ranged from 0.10% to 8.08%, with highest reported prevalence values found in Africa including Cameroon. In Cameroon, podoconiosis was first described in 1981 by Dr Ernest Price (Price & Henderson, 1981). In 2018, a nationwide study indicated an overall prevalence of 0.5% with about eight health districts presenting with mean prevalence between 1.2% and 2.7% (Deribe et al., 2018b)

Leprosy (Hansen's disease), is a chronic infectious disease caused by a slow-growing bacterium called *Mycobacterium leprae* (*M. leprae*). In 2017, WHO reported 171 948 cases as receiving MDT (Multidrug therapy), with a prevalence rate of 0.23 per 10 000 populations from 143 countries (World Health Organization, 2016). In 2016, 214 783 new cases were reported from 143 countries with a global new-case detection rate of 2.9 per 100 000 populations (World Health Organization, 2016). Between 2000 and 2014, Cameroon recorded a significant drop in leprosy prevalence, detection rates and overall burden (Tabah et al., 2016). In 2000, leprosy elimination was declared in Cameroon even though a good number of health districts remain high-leprosy-burdened including Essimbiland and Mbingo in the North West Region (Nsagha et al., 2014). Reasons for such high prevalence could be attributed to geo-cultural characteristics and tribal belief systems regarding leprosy as a spell which can only be treated by traditional healers (Tabah et al., 2016).

Untreated, leprosy and podoconiosis progresses to result in damage of the affected areas causing devastating disfigurement and disability leading to social stigma and isolation of affected persons and their family members (Deribe et al., 2013; Laza & Codreanu, 2018; Mousley et al., 2013; Tora et al., 2014). Currently, there exist no national health system plans or strategy for podoconiosis control, even though, efforts have been made by local NGOs in partnership with international bodies to alleviate suffering through community and health facility-based interventions. On the other hand, a well-coordinated National Leprosy Control Program (NLCP) exist with rehabilitation and treatment centers which aids in alleviating leprosy related stigma and isolation in endemic communities (WHO, 2005).

Both diseases affect quality of life physically, socially and economically through pain, disability, reduced productivity, marginalization, stigma, difficulties in finding employment, gaining education and getting married (GebreHanna, 2005; Mousley et al., 2013; Tembei et al., 2018; Tora et al., 2014; Van Brakel, 2003; WHO, 2001). These are vital activities for both social and economic well-being (WHO, 2009). Stigma can be divided into enacted stigma and felt stigma (Franklin et al., 2013; Link & Phelan, 2006; Weiss et al., 2006). Prejudice and discrimination against patients by family members exposes them to deprivation of emotional and material support (Fikresilasie, 2015; Tora et al., 2011).

Despite its prevalence and socioeconomic burden, podoconiosis remains one of the most neglected of Neglected Tropical Diseases in Cameroon while leprosy has gained adequate public health attention. There is much less information available on the social burden of podoconiosis compared to leprosy. The inadequate public health attention to podoconiosis may be due to this paucity of research-based evidence to inform policy decisions in the country. To close this gap, this study seeks to assess and compare the level of similarity in podoconiosis and leprosy related quality of life and stigma at different levels namely patients, family members of patients and neighbours of affected households. The study therefore aims to explore and document the existence of podoconiosis related stigma and quality of life issues in Cameroon while comparing it to that of well-known stigmatizing leprosy.

## 2 Methods

### 2.1 Study area

The North West Region of Cameroon has 19 Health Districts with the Batibo and Ndop Health Districts being among the most affected with podoconiosis (Wanji et al., 2008). Most of the population of Batibo and Ndop practice subsistence agriculture, which is the main economic driver within both communities. The Ndop plain is known in the region for the cultivation of rice in marshy wetlands. These agricultural factors predispose the inhabitants to acquiring podoconiosis due to continuous direct and indirect exposure of bare feet to the soil. The Mbingo Baptist Leprosarium, owned by the Cameroon Baptist Convention, a missionary group, is one of the oldest leprosaria in Cameroon, and is found in the Fundong Health District within the Mejang Health Area. Many households with a leprosy resident are found in this area, due to referrals from other regions of the country. Data collection spanned from July to August 2015.

### 2.2 Study design

**Fig. 1:**
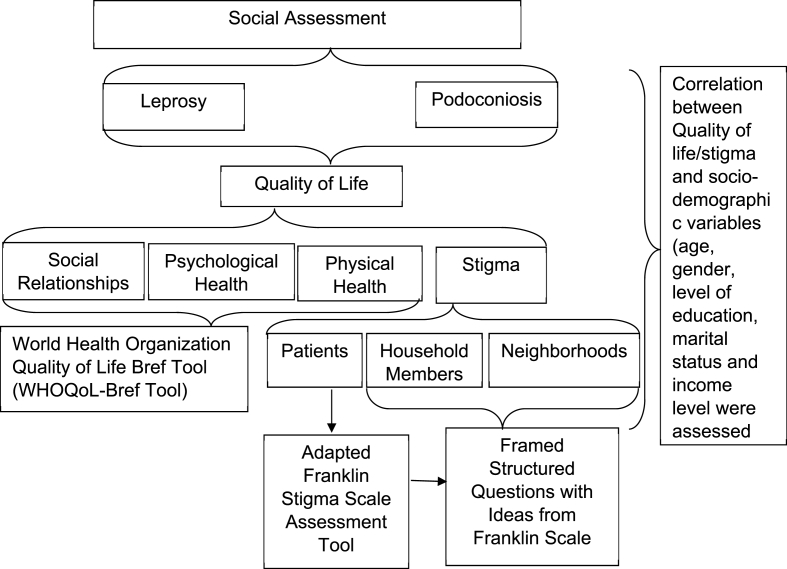
A cross sectional study design for assessing the quality of life/stigma of Podoconiosis and Leprosy affected households.

A questionnaire-based comparative cross sectional study design was used in this study (see Fig. 1).

Two distinct groups were used for the comparison: the first group was composed of clinically confirmed podoconiosis patients and the second group composed of clinically confirmed leprosy cases. Household members of the case persons and one neighbor household within each case neighborhood were included in the study to assess stigma perceptions from these groups of persons.

### 2.3 Sample size, sampling and study subjects

Given the small nature of this study which was implemented only in three health districts of the North West Region coupled with the sparse distribution of podoconiosis disease in Cameroon, Epi Info version 7.0 was used to calculate a minimum sample size for cross-sectional studies. The formula assumed that 50% of podoconiosis patients in the study population experience low quality of life (as suggested for studies with unknown disease prevalence (Martínez-Mesa et al., 2014)), 80% power to detect a 30% difference quality of life between the two groups at 95% confidence interval. This yielded a minimum sample size of 39 patients per disease group summing up to a total of 78 patients. One household member within a case household (a total of 86) and 1 neighbor member (a total of 86) from a neighbor household were envisaged to be interviewed.

Participants included podoconiosis and leprosy patients 15 years and more. Podoconiosis have been revealed to be most common within the economically active age group from 15 years and above due to the duration of time before onset or manifestation. This age-group equally represents the most affected for leprosy. Based on the study design and for comparison purposes, both groups of patients were limited to this age group. One family member (defined as anyone aged 21 years (legal age for consent by Cameroon law) and above living with the case person under the same roof) within each podoconiosis and leprosy case household and one neighbor from a neighbor household (defined as a household living in the same quarter (small geographical sub-populations that make up a community) within the community of the case household with a member matching the case person's age (±5years), gender and occupation) within each case neighborhood were interviewed. Age, gender and income have been shown to be strongly associated with levels of quality of life and stigma (Hofstraat et al., 2016; Mousley et al., 2013). Neighborhood was limited to the same quarter where the case household lives without any notion of distance due to difficulty in finding matched cases within the recommended age, gender and occupation. A quarter represents traditionally delineated geographical sub-populations within the same community. The traditional boundaries delineated by the community to represent a quarter were respected. An age band ±5 years was considered to widen the scope of having a match control since ±2 years was very challenging to find within the same quarter. For easy identification of control households in the quarter, case households were requested to provide control households within the quarter that fulfils the above criteria. In cases where more than one control households were identified, the household with the closest proximity to the case household was selected for interview. In cases where the selected household was absent, the next available household meeting the criteria was selected. Interviews in control households targeted either the household head or the most knowledgeable and eligible household member and not necessarily the matched person.

Podoconiosis patients living in households within Batibo and Ndop health districts were sequentially sampled using a list of confirmed podoconioisis cases from previous studies (Wanji et al., 2008, 2018).

Leprosy patients (currently or previously on treatment) living within communities in Mejang Health Area were sequentially sampled using the Mbingo treatment center register. The Mbingo treatment center has 2 leprosy camps. The New Hope camp consisting of active leprosy resident cases on treatment and Camp consisting of previously treated leprosy resident cases. Other previously treated leprosy households were found in quarters around the treatment center. Leprosy patients referred to the treatment center from other areas in Cameroon and admitted at the leprosy ward were excluded from the study due to the study design which required interviewing neighbours of patients within their local resident communities. A total of 18 households with an active leprosy patient were identified and interviewed from the New Hope camp. Thirteen households with a previously treated leprosy patient were interviewed from the New Hope quarter, 11 from Camp and 1 from another quarter around the center.

### 2.4 Variables and study instruments

Variables of interest included, quality of life scores of patients, felt and enacted stigma scores of patients, stigma scores of family members and stigma scores of neighbours. A quantitative methodology was employed adapting quality of life and stigma standardized structured questionnaires. Method of assessment for all variables of interest for both disease households and neighbor households were the same. It is worth mentioning that the scope of this study was limited to 4 quality of life domains namely; physical health, psychological health, social relationships and stigma. Other quality of life domains such as environmental health and mental health domains were not captured in this study due to their broad nature which was beyond the scope of this study.

The WHOQoL-BREF scale was developed from the WHOQOL 100 scale and assesses quality of life in 4 domains. Details about the development and psychometric properties of the tool have previously been documented (World Health Organization, 1998). The WHOQoL-BREF tool was adapted to assess quality of life in three domains namely; physical health (4 items), psychological health (4 items) and social relationships (3 items). WHOQoL-BREF uses a 5-point scale for each answer, and these are scored positively, with higher values meaning a higher quality of life. In this study, higher values meant low quality of life. Scores for negatively framed questions were reversed before calculating scores for each domain. Raw domain scores were calculated as a mean of all domain items multiplied by 4. Raw domain scores were then transformed on a scale from 0 to 100 as described by World Health Organization (World Health Organization, 1998).

### 2.5 Stigma instrument

The Franklin stigma scale was used to assess felt and enacted stigma among podoconiosis patients. The Franklin Stigma scale was developed using items that measured stigma related to leprosy due to its high stigmatizing attributes with symptoms that are hard to conceal, like podoconiosis. Since individuals with podoconiosis face similar exclusionary social treatment to those with leprosy, commonly used items in leprosy stigma assessments were included in developing the Franklin stigma scale (Franklin et al., 2013).

Given the nature and scope of this study being an exploratory study to rapidly demonstrate the existence of podoconiosis related stigma in Cameroon and comparing it to already existing stigmatizing leprosy, items for patients’ felt and enacted stigma on Franklin stigma scale was adapted and modified, hence, different number of items were assessed in this study as compared to the number of items on the original stigma scale. Some items perceived to have close related ideas on the original scale were merged in this study for rapid assessment resulting in lesser number of items on the current scale. Felt stigma for patients was assessed based on seven items while enacted stigma had five items, all based on the indicators in Table 1.

**Table 1:** Indicators of disease stigma in three domains.

DOMAIN 1 Interpersonal interactions (domestic life, family/neighborhood relationships)	DOMAIN 2 Major life areas	DOMAIN 3 Community, social and civic life
Buying items at market	Employment	Leadership and decision making
Eating/living separately	Marriage	Participation in community affairs, public events and social organizations
Interactions with family, friends, neighbours and health professionals		
Isolation from others		
Shame/embarrassment		

In order to capture the perceptions and views of unaffected household and community members, concepts from the Franklin stigma scale were used to frame questions to be answered by household members and neighbours. Ideas on the kind of stigma concepts to ask from family members and unaffected community members were gotten from the Franklin scale which aid in the development of questions for unaffected household members and neighbours in this study. This was done to either confirm or contradict the level of patient reported felt and enacted stigma by understanding the views of household members and neighbours. Perceptions of stigma from household members and from neighbours were explored using eight items without categorizing them as either being felt or enacted.

A 1-point dichotomous scale was used for assessing stigma using 0 for No and 1 for Yes. Negatively framed questions were inverted before analyzing. Item scores per stigma domains were summed. The stigma scores for each domain were converted on a 0–100 scale. To convert domain scores on a 0–100 scale, the maximum score an individual could obtain per domain was multiplied by a factor which will yield 100. Overall stigma scores for each level of assessment were calculated as a median of all domain scores. Higher stigma scores meant higher stigma perceptions in this study.

The study instruments for QoL and stigma were constructed in English language and administered either in English language where appropriate or “Pidgin English” which is the most common local language being spoken in rural communities with little or no educational training in the North West Region. An instrument pre-test was done to test the practicality of the instrument within the context of the study. Following pre-testing, a final instrument was developed with some questions being re-positioned and others rephrased for clarity and to reduce time taken to complete an interview session which was approximately 30 min. Also, agricultural products for the communities were identified and incorporated into the instrument to estimate household economic productivity.

### 2.6 Data collection procedure

Interviewers constituted Masters students in epidemiology from the University of Buea fluent in Pidgin English who were familiar and had experience in community-based research approaches involving NTDs. They were trained on the study instruments, the diseases, manner of approach in households and techniques of interview.

Podoconiosis patients were traced into their communities and interviewed at their households. Leprosy patients within the camps were approached at their households using the Mbingo leprosy health personnel. One unaffected household within the neighborhood with an individual matching the case person by age (±5years), gender and occupation was identified for interview following the aforementioned sampling criteria.

All interviews were carried out within the household premises. Patients were interviewed individually away from family members. Interview for family members and neighbours targeted the household head or most knowledgeable household member who was interviewed away from other household members. Issues regarding co-morbidity were considered during the interview process. Efforts were made to minimize any confounding stigma reports drawing from other diseases as questions were repeatedly prompted for responses specific to the disease of interest all through the data collection process.

Household yearly income was estimated based on yearly agricultural productivity and yearly salary for households on monthly payment. Household earnings were compiled with the household providers and was calculated based on the combine earnings of all household members for the period of one month extrapolated to a calendar year. Based on this, employed household members were asked to estimate their monthly salaries. Unemployed household members who relied on agricultural products for income generation were asked to quantify their seasonal products per year. These quantities were then multiplied by the average unit market price per commodity within the community and then, summed with monthly salaries to get yearly household income. Socio-demographic variables like age, gender, income status, marital status, level of education and years lived in community for patients only were assessed to verify their association of some variables with quality of life and stigma.

### 2.7 Data analysis

Variables were pre-coded and data were entered into Epi-Info v.3.5 (CDC, Atlanta, GA, USA); and imported to SPSS v. 20 (IBM Corp., Armonk, NY, USA) and Stata v. 15 for analysis. Tables were used to describe data. Statistical significance of the differences in socio-demographic characteristics of patients between disease groups were assessed using the chi^2^ test, *t*-test and Mann-Whitney *U* test where appropriate. Reliability of the questionnaire used was assessed through consistency analysis using Cronbach's alpha. Validity was analyzed using exploratory factor analysis (FA) with principal components analysis (PCA). Following skewness and kurtosis normality test, differences in the distribution of QoL and Stigma scores between disease groups were compared using the Mann-Whitney *U* test which test the comparability of distribution across groups when data is not normally distributed. Households with missing data for variables of interest at each level of assessment were excluded during analysis. For QoL and patient felt and enacted stigma, median scores of the patients were presented. For stigma from family members against patients, median scores of family members were presented. For stigma from neighborhoods, median scores of neighbours were presented. Due to the non-normality of the distribution, quantile regression was used to estimate the linear relationship existing between each outcome variable (quality of life domains and stigma) and socio-demographic variables around the 50 quantile while controlling for covariates.

## 3 Results

### 3.1 Basic characteristics of patients

A total of 79 podoconiosis households were identified from 12 health areas in Batibo (09) and Ndop (03) health districts. Thirty-six households could not be reached either due to road inaccessibility or could not be traced in the community by the research team. Forty-five leprosy households were identified through the leprosarium. Two households could not be interviewed. One household was absent (occupants had travelled for holiday) and the other household had no eligible household member to provide consent on behalf of an adolescent leprosy child.

A total sample of 86 patient households (43 podoconiosis and 43 leprosy patients) were recruited for this study with each household yielding a single case. Response rate for all households with case person available for interview was 100%. This was due to the fact that, podoconiosis households were already familiar with the research team under which this project was conducted from previous studies. Leprosy households were approached through the leprosarium. The chief of service formally informed all patients within the camp about the study, requested full collaboration and provided a guide whom the patients were familiar with to aid the process.

Of the 86 patient households available for interview, 2 households (1 podoconiosis and 1 leprosy) were excluded from the analysis due to inability to respond to the interview as a result of ill health of the patient and no eligible household member. Hence, the socio-demographic information of 84 case patients have been presented (Table 2). Sex was evenly distributed between males and females per disease group, and mean age among patients was 59 and 60 years for podoconiosis and leprosy respectively. Most of the patients were either married or widowed.

**Table 2:** Basic characteristics of participants.

Variable	Category	Podoconiosis Cases (%)	Leprosy Cases (%)	P-Value
**Gender**	Male	21 (50)	21 (50)	1.000 ^δ^
	Female	21 (50)	21 (50)	
**Age**	Mean (SD)	59 (16.8)	61 (16)	0.729 (95% CI: −5.840 – 8.316)^b^
	Range	57 (30–87)	60 (27–87)	
**Level of Education**	Higher	3 (75.0)	1 (25.0)	0.737^a^
	Secondary	4 (57.1)	3 (42.9)	
	Primary	22 (47.8)	24 (52.2)	
	None	13 (48.1)	14 (51.9)	
**Marital Status**	Single	3 (23.1)	10 (76.9)	0.082 a
	Married/In Union	22 (61.1)	14 (38.9)	
	Divorced/Separated	3 (33.3)	6 (66.7)	
	Widowed	14 (53.8)	12 (46.2)	
**Household Income (USD)**	Median (IQR)	388 (130–802)	324 (121–1254)	0.690 (U: 1428, Z = −0.403)^c^
**Religion**	Christian	42 (50)	42 (50)	NA

a Chi-square test.

b *t*-test.

c Mann-Whitney *U* test; CI: Confidence Interval; IQR: Interquartile Range; NA: Not Applicable; %: Percentage; SD: Standard Deviation; USD: United States Dollars (exchange rate September 10, 2015: US$1 = 586FCFA).

### 3.2 Quality of life

The psychometric properties of the QoL instrument showed good consistency with Cronbach's alpha of over 0.7 for overall QoL and physical health domains while moderate consistency was observed for psychological health and social relationship domains with alpha of 0.6. None of the QoL domains had inter-item correlation greater than 0.9. Factor analysis for all QoL domains showed Kaiser-Meyer-Olkin measure of sampling adequacy above 50% with significant Bartlett's test of sphericity at p < 0.05. With factor loadings of more than 0.4, all QoL domains extracted 01 component with eigenvalue greater than or equal to unity. These results indicated satisfactory reliability and validity of the tool used in measuring quality of life.

Comparing QoL domain scores between leprosy and podoconiosis patients, only the physical domain showed a significant difference in the distribution of scores between both disease groups with p < 0.05, median: 70 (IQR: 55–75); U: 635 and effect size (r) of 0.2 (Table 3). No differences in the distribution of scores between groups were observed for the psychological and social relationship domains.

**Table 3:** Quality of life assessment in three domains.

Quality of Life Domains	N (42 Podo, 42 Leprosy)	Median (IQR)	Mann-Whitney U	Z	Effect size (r)	P-value (5% significance level)
Physical Health	84	70 (55–75)	635	−2.2	0.2	0.025*
Psychological Health	84	63 (55–70)	816	−0.6	0.1	0.550
Social Relationships^#^	83	60 (53–67)	836	−0.2	0.0	0.817

^a^*Significant at p < 0.05.

^b^Scores on a scale of 0–100.

^c^ IQR: Interquartile Range.

^d^ Z: Z-statistics.

^e^^#^N = 41 for Podo (Podoconiosis).

### 3.3 Stigma related to podoconiosis and leprosy patients

The psychometric properties of the stigma instrument showed good consistency with Cronbach's alpha of 0.7 for patient enacted stigma, family members and stigma from neigbours scales. None of these stigma scales had inter-item correlation greater than 0.9. Factor analysis for stigma scales showed Kaiser-Meyer-Olkin measure of sampling adequacy above 50% with significant Bartlett's test of sphericity at P < 0.05. With factor loadings of more than 0.4 considered as being satisfactory, the principal component analysis extracted at least 2 components with eigenvalue greater than or equal to unity. These results indicated satisfactory reliability and validity of the tools for patient enacted stigma, stigma from family members and stigma from neigbours. Patient felt stigma scale showed evidence of multi-collinearity between 2 items. Consequently, no further analysis was performed on patient felt stigma.

### 3.4 Patients enacted stigma

All patient enacted stigma domains had median scores of 100 (IQR: 100-100) for both leprosy and podoconiosis. The test for similarity in the distribution of stigma scores revealed significant variation in the major life area domain and overall enacted stigma at p < 0.05 and effect size of 0.3 and 0.4 respectively (Table 4).

**Table 4:** Patients enacted stigma.

Stigma Domains	N (42 Podo, 42 Leprosy)	Median (IQR)	Mann-Whitney U	Z	Effect size (r)	P-Value (5% significance level)
Interpersonal Interactions	84	100 (100–100)	762	−1.8	0.2	0.112
Major Life Area	84	100 (100–100)	714	−2.7	0.3	0.015*
Community, Social and Civic Life	84	100 (100–100)	756	−2.0	0.2	0.088
Overall Stigma	84	100 (100–100)	594	−3.5	0.4	p < 0.001*

*Significant at p < 0.05; IQR: Interquartile range; Z: Z-statistics; Scores on a scale of 0–100; Podo: Podoconiosis.

### 3.5 Stigma related to household members and neighbours of podoconiosis and leprosy patients

The number of neighbor households interviewed for podoconiosis cases were 38 and 39 for leprosy cases. This was due to no neighbour households with a match control corresponding to the case person's age, sex and occupation around some case households. The number of podoconiosis case households with family member's interviewed were 41 and 22 for leprosy. This was due to the fact that, for some case households, there were no eligible household members who could respond to the interview either because the case person lived alone or with one or two minors as their care takers.

When comparing stigma related to family members either as a results of having a patient at home or being related to a patient from community perspective, only overall stigma had a significant difference in the distribution of scores for both leprosy and podoconiosis households at p < 0.05, median:17 (IQR: 0–15), U: 627 and effect size (r) of 0.3 (Table 5).

**Table 5:** Stigma related to family members and neighbours of podoconiosis and leprosy patients.

Stigma Domains	N (42 Podo, 42 Leprosy)	Median (IQR)	Mann-Whitney U	Z	Effect size (r)	P-Value (5% significance level)
**Stigma from Family Members**						
Interpersonal Interactions	62 (41 podo; 21 leprosy)	25 (0–50)	674	−2.0	0.2	0.050
Major Life Area	60 (38 podo; 22 leprosy)	0 (0–100)	374	−0.8	0.1	0.589
Community, Social and Civic Life	59 (37 podo; 22 leprosy)	0 (0–50)	311	−1.7	0.2	0.103
Overall Stigma	62 (40 podo; 22 leprosy)	17 (0–50)	627	−2.3	0.3	0.018*
**Stigma from Neighbours**						
Interpersonal Interactions^#^	76	50 (25–75)	446	−2.9	0.3	0.003*
Major Life Area^#^	76	50 (50–100)	512	−2.5	0.3	0.014*
Community, Social and Civic Life ^#^	76	50 (50–100)	306	−5.0	0.6	p < 0.001*
Overall Stigma ^#^	76	67 (42–75)	336	−4.0	0.5	p < 0.001*

* Significant at p < 0.05; podo: podoconiosis; ^#^ = 37 for podo & 39 for leprosy; IQR: Interquartile range; Scores on a scale of 0–100.

For stigma from neighbours, all stigma domains including overall stigma (median: 67 (IQR: 42–75), U: 336 and effect size (r) of 0.5) showed significant differences in the distribution of scores for both leprosy and podoconiosis neighbours at p < 0.05 (Table 5).

### 3.6 Association between QoL/Stigma and patients socio-demographic variables for podoconiosis affected households

A quantile regression analysis verifying linear relationship between the median scores for quality of life domains, overall stigma from family members and neighbours with socio-demographic characteristics of patients such as gender, age, income status, education and marital status was performed. The results presented show the relationship at the 0.5 quantile. Results at the 0.25 and 0.75 quantiles are presented under the appendix section. Overall patients enacted stigma showed limited variation around the median, hence regression analysis was not applicable.

With respect to QoL relationship, annual income was observed to have regression coefficients equal to zero with a significant non-linear relationship across all quality of life domains for leprosy patients and a non-significant non-linear relationship for podoconiosis patients at p < 0.05, 95% CI (Table 6). Compared to other socio-demographic variables, sex was observed to have the highest effect in terms of magnitude on physical (B = −7.938; p = 0.078 [−16.825–0.95]) and psychological (B = −5.942; p = 0.234 [−15.891–4.006]) health domains for podoconiosis and physical (B = −4.486; p = 0.509 [−18.11–9.138]) and social relationships (B = −5.194; p = 0.349 [−16.297–5.908]) domains for leprosy patients with p > 0.05, 95% CI.

**Table 6:** Relationship between quality of life and socio-demographic variables for podoconiosis and leprosy patients.

Dependent Variable	Independent Variable	Podoconiosis				Leprosy			
		B	p-value	[95% Conf Interval]		B	p-value	[95% Conf Interval]	
Physical Health	Sex	−7.938	.078	−16.825	.95	−4.486	.509	−18.11	9.138
	Age	.094	.49	−.179	.366	.286	.242	−.202	.775
	Level Of Education	−.923	.781	−7.622	5.776	.817	.891	−11.183	12.818
	Marital Status	1.219	.57	−3.098	5.535	1.857	.534	−4.143	7.857
	Annual Income (USD)	0	.623	0	0	0	.025*	0	0
Psychological Health	Sex	−5.942	.234	−15.891	4.006	−3.722	.444	−13.486	6.041
	Age	−.152	.319	−.457	.153	−.337	.059	−.686	.013
	Level Of Education	−2.244	.548	−9.743	5.255	−2.694	.529	−11.294	5.906
	Marital Status	.157	.948	−4.675	4.989	.313	.883	−3.987	4.613
	Annual Income (USD)	0	.72	0	0	0	.037*	0	0
Social Relationships	Sex	−.046	.989	−6.582	6.489	−5.194	.349	−16.297	5.908
	Age	−.001	.989	−.204	.201	−.233	.243	−.631	.165
	Level Of Education	.117	.962	−4.791	5.026	−4.385	.369	−14.164	5.395
	Marital Status	−.058	.971	−3.269	3.153	.424	.861	−4.465	5.314
	Annual Income (USD)	0	.97	0	0	0	.037*	0	0

*Significant at the 0.05 level (2-tailed).

For stigma relationships, annual income was equally observed to have regression coefficients equal to zero for both family members and neighbours overall stigma. A significant non-linear relationship (p = 0.024 [0-0) was observed between annual income for overall stigma from family members for leprosy (Table 7). Age showed a negative but significant relationship (B = −1.113; p = 0.01 [−1.948 to −0.278) with stigma from podoconiosis family members. Sex still revealed highest effect on overall stigma for both family members and neighbours for podoconiosis with p > 0.05. For leprosy, sex had no significant effect for family members (B = 12.326; p = 0.324 [−12.681–37.334]) while level of education had highest significant effect on overall stigma for neighbours (B = −8.696; p = 0.454 [−32.063–14.67]).

**Table 7:** Relationship between stigma and socio-demographic variables for podoconiosis and leprosy family members and neighbours.

Dependent Variable	Independent Variable	Podoconiosis				Leprosy			
		B	p-value	[95% Conf Interval]		B	p-value	[95% Conf Interval]	
Overall Stigma from Family Members	Sex	−24.004	.083	−51.263	3.255	12.326	.324	−12.681	37.334
	Age	−1.113	.01*	−1.948	−.278	.42	.348	−.476	1.316
	Level Of Education	−4.267	.676	−24.814	16.279	5.039	.645	−16.988	27.067
	Marital Status	−5.532	.402	−18.771	7.708	−.14	.98	−11.153	10.872
	Annual Income (USD)	0	.862	0	0	0	.024*	0	0
Overall Stigma from Neighbours	Sex	−16.826	.213	−43.8	10.149	.039	.997	−24.848	24.925
	Age	−.021	.959	−.849	.807	−.218	.63	−1.128	.693
	Level Of Education	−.215	.982	−19.701	19.27	−8.696	.454	−32.063	14.67
	Marital Status	8.233	.218	−5.134	21.6	−1.247	.816	−12.082	9.588
	Annual Income (USD)	0	.833	0	0	0	.715	0	0

*Significant at the 0.05 level (2-tailed).

## 4 Discussion

For the first time, we have demonstrated almost equivalent levels in the distribution of quality of life and stigma among podoconiosis and leprosy patients. For podoconiosis and leprosy patients, the physical health showed significant differences in the distribution of quality of life with podoconiosis patients having higher probability of experiencing lower quality of life than leprosy patients. Psychological health and social relationship showed that podoconiosis patients had lower chances in experiencing low QoL in these aspects of life compared to leprosy patients. These findings imply that, both podoconiosis and leprosy patients experience similar, if not the same quality of life when looking at their psychological health and social relationship activities. The similarities observed in QoL perceptions between disease groups could mean that, podoconiosis and leprosy patients in this study turn to express similar un-satisfaction in their quality of life standards. Similar findings have been documented from studies in India, Brazil, Ethiopia and Côte d'Ivoire where leprosy and podoconiosis patients were shown to experience significant lower average QoL compared to unaffected persons. These studies highlighted that podoconiosis exerted greater impacts on patients QoL compared to other NTDs such as schistosomiasis or similar challenges in physical health compared to helminthiasis and malaria patients (Fürst et al., 2012; Joseph and Sundar Rao, 1999; Mankar et al., 2011; Mousley et al., 2013; Reis et al., 2013). Drawing from reports of other studies in literature as seen in the above examples, the comparability in the distributional of QoL scores across domains for both diseases in this study, express to an extent that podoconiosis patients in Cameroon like leprosy patients experience low QoL.

### 4.1 Stigma related with podoconiosis and leprosy

This study reported high median scores (indicating high stigma levels) for enacted stigma. Overall, high enacted stigma scores were observed for leprosy and podoconiosis which was constant across all three stigma domains. This study revealed that leprosy patients’ had higher probability in experiencing enacted stigma compared to podoconiosis patients and this was greatest for the major life area domain and overall stigma. According to the WHO International Classification of Functioning, Disability and Health (ICF) major life area was one of the areas reported to have greatest impact for leprosy stigma (Van Brakel, 2003; WHO, 2001). Studies in Ethiopia have reported high levels of enacted stigma and demonstrated the effect podoconiosis related stigma has on mobility, domestic life, interpersonal interactions and relationships, major life areas, and community, social and civic life of affected persons (GebreHanna, 2005; Tora et al., 2011, 2014).

This suggests that podoconiosis patients in Cameroon just like leprosy patients do experience prejudice and discrimination shown to them by the community. Stigma related to family members either as a results of having a patient at home or being related to a patient from community perspective, was shown to be comparable for all stigma domains and overall stigma for both podoconiosis and leprosy households. Compared to family members from leprosy households, those from podoconiosis households turn to have higher chances of being stigmatized in the interpersonal interactions, community, social and civic life domains and overall stigma. Leprosy households experience considerably significant greater levels of stigma from their neighbours in all stigma domains and overall stigma compared to podoconiosis households. These findings suggest that podoconiosis household members overestimate the stigma coming from their community members against them while leprosy households reported stigma levels borne out by their neighbours. Studies in Ethiopia revealed that more than one-half of their participants showed stigmatizing attitudes towards podoconiosis (Yakob et al., 2008). The difference observed between perceptions of stigma from neighbours towards leprosy and podoconiosis affected households may be attributable to the disease stage of most podoconiosis cases. Although not captured within the scope of this study, stigma associated with podoconiosis appears to increase with increase in the stage of the disease (Deribe et al., 2013; Tora et al., 2014). Patients with disease stage three and above are more stigmatized than patients below stage three. Similarly, disease stage had been found to be positively associated with stigma among elephantiasis patients due to Lymphatic Filariasis (Kumari et al., 2005; Person et al., 2007).

### 4.2 Association between QoL/Stigma and socio-demographic variables of patients

All predictor variables with an exception of annual income were observed to have a non-significant linear relationship with quality of life of leprosy and podoconiosis patients across all domains. The non-linear relationship revealed between annual income and QoL for both diseases could imply that variables such as sex, age, education and marital status play a vital role in the way income affects quality of life of both patients (Develarist, 2020).

Age of podoconiosis patients was observed to have a significant negative linear relationship with stigma from podoconiosis family members against podoconiosis patients. This implies that, a change in age of a podoconiosis patient has a predictive stigma effect of 1.11 from family members. Studies have equally reported income to have significant effects on leprosy QoL as well as the effect of sex on leprosy (Joseph and Sundar Rao, 1999; Lustosa et al., 2011; Tsutsumi et al., 2007). A study on social stigma of leprosy in Nepal by Marahatta et al. showed that both men and women faced the social stigma of the disease, although women were revealed to suffer more rejection by the family members, neighborhood and work places compared to men (Marahatta et al., 2015). This report is consistent with our finding revealing high magnitude of association between leprosy stigma and gender for family members.

### 4.3 Limitations

Given the strength of this study in comparing and quantifying for the first time the quality of life and stigma related to podoconiosis and leprosy diseases in Cameroon, the study was limited due to the small sample size and hence, low statistical power. The authors recommend that a further study with larger sample size be carried out to better generalize these findings. The study considered three rather than four WHOQoL domains, which may have compromised ability to effectively compare with other studies. Due to the fact that some questions were merged from the original stigma scale, the degree of some stigma concepts may have been concealed and under reported or completely lost out from the assessment. The patient felt stigma could not be assessed as planned due poor reliability and validity of the patient felt stigma scale. The study recommends more research be conducted in the country on this aspect of stigma. QoL and stigma reported in this study was limited to age group 15 years and above. Hence, no picture of the situation among lower age groups is revealed. Future studies should consider this age groups. Random selection was not practically possible due to the wide distribution of podoconiosis inhabitants within the study districts, and so sequential sampling was used, with the first podoconiosis patients on patient list who could be found being selected. Given the low literacy level of the population, data collection was interview-based and not self-administered; we acknowledge that this may have reduced the reliability of the findings. More so, the interview did not consider men to men and women to women interviewer vs. interviewee. This could affect the willingness of patients to expose certain gender related perceptions. The recruitment of leprosy patients through Mbingo treatment center may have introduced selection bias, possibly resulting in exclusion of the most stigmatized leprosy patients who may not have reached the treatment center. Information on the state of treatment for leprosy participants and the disease stage of podoconiosis participants at the time of data collection was not captured. Although not captured, it is worth noting that majority of podoconiosis cases sampled where between the stages 2–3. Perception of stigma related to household members and neighbours were assessed without distinguishing them as either being felt or enacted. This can conceal the degree of either felt or enacted stigma reported or experienced by these groups of people. Socio-demographic characteristics of household members and neighbours were not captured in this study; only the characteristics for patients were captured and presented.

## 5 Conclusions

First, this study has successfully demonstrated the existence of podoconiosis related stigma among patients in Cameroon. Secondly, the study confirmed the hypothesis that people with podoconiosis experience low quality of life just like those affected with leprosy. Thirdly, the study has successfully demonstrated the comparability in high stigma levels being experienced by podoconiosis and leprosy patients in the country. Finally, this study illustrated that unaffected household members and neighbours hold high levels of stigma against both groups of patients or stigma coming from the community towards these unaffected members due to their relationship with the affected persons. These findings add evidence to already existing knowledge on the current burden of podoconiosis in Cameroon in terms of prevalence, clinical manifestation and associated morbidity, and treatment cost burden (Deribe et al., 2018b; Hofstraat et al., 2016; Tembei et al., 2018). These evidences put together build a great reservoir of knowledge to inform public health actions. There is a great need to leverage public health interventions such as case management, rehabilitation and social services in endemic rural areas all in a bit to prevent and control podoconiosis related morbidity.

## Funding

This research did not receive any specific grant from funding agencies in the public, commercial, or not-for-profit sectors.

## Ethical consideration

Ethical approval was obtained from the University of Buea Faculty of Health Science Institutional Review Board (Ref: 2015/368/UB/FHS/IRB) and Bamenda Regional Hospital Institutional Review Board after a Departmental Authorization was issued from the University of Buea (Ref: 2015/263/UB/FS/HOD/MBP). Regional and Administrative Clearances were obtained from the North West Regional Delegation of Public Health (No: 1004/1/NWR/RDPH) and from District Medical officers of Health Districts. Permission was also obtained from the Cameroon Baptist Convention Health Service (Ref: CBC/DHS-L/15/24 506) to collect data at the Mbingo Baptist Hospital. Written informed consent forms were used to ensure participants’ willingness to participate in the study using methods described by Kengne-Ouafo et al. (Kengne-Ouafo et al., 2014)

## Authors statement

SW and AMT conceived the study; SW, AMT, TNA, PE, TMN and JAKO designed the study protocol; AMT, TMN, and BJ conducted the interviews; AMT, TMN, JAKO, and SW analyzed and interpreted the data; AMT, SW, JAKO and TMN drafted the manuscript; AMT, SW, GD, TNA, PE, JAKO, TMN and BJ critically revised the manuscript for intellectual content; AMT, JAKO, TMN, BJ, PE, TNA, GD and SW read and approved the final manuscript. SW is the guarantor of the paper.

## Declaration of competing interest

None declared.

## Figures and Tables

**Table:** 

0.75 Quantile Overall Stigma from Family Members and Neighbours									
Dependent Variable	Independent Variable	Podoconiosis				Leprosy			
		B	p-value	[95% Conf Interval]		B	p-value	[95% Conf nterval]	
Overall Stigma from Family Members	Sex	−31.681	.141	−74.392	11.03	8.622	.602	−24.641	41.885
	Age	−.774	.238	−2.083	.534	.521	.381	−.671	1.712
	Level Of Education	4.344	.786	−27.849	36.537	−16.456	.262	−45.756	12.844
	Marital Status	−12.491	.23	−33.236	8.253	−2.688	.712	−17.336	11.961
	Annual Income (USD)	0	.84	0	0	0	.005*	0	0
Overall Stigma from Neighbours	Sex	−26.852	.061	−55.016	1.313	−4.427	.62	−22.414	13.561
	Age	−.414	.336	−1.279	.45	−.467	.159	−1.125	.191
	Level Of Education	−16.947	.099	−37.291	3.398	1.899	.82	−14.989	18.788
	Marital Status	5.493	.428	−8.463	19.45	7.302	.067	−.529	15.134
	Annual Income (USD)	0	.693	0	0	0	.081	0	0
**0.25 Quantile Overall Stigma from Family Members and Neighbours**									
Overall Stigma from Family Members	Sex	−11.101	.299	−32.447	10.245	2.488	.512	−5.129	10.105
	Age	−.126	.698	−.78	.528	.17	.214	−.103	.443
	Level Of Education	3.691	.645	−12.399	19.78	1.548	.643	−5.162	8.257
	Marital Status	−5.959	.251	−16.327	4.408	.459	.783	−2.895	3.813
	Annual Income (USD)	0	.882	0	0	0	p < 0.001	0	0
Overall Stigma from Neighbours	Sex	−19.376	.068	−40.238	1.486	10.637	.439	−16.997	38.271
	Age	.264	.407	−.376	.904	.107	.832	−.905	1.118
	Level Of Education	6.132	.413	−8.938	21.201	−8.921	.489	−34.867	17.025
	Marital Status	6.767	.192	−3.571	17.105	−.903	.88	−12.934	11.129
	Annual Income (USD)	0	.728	0	0	0	.371	0	0

*Significant at the 0.05 level (2-tailed).

**Table:** 

0.75 Quantile									
Dependent Variable	Independent Variable	Podoconiosis				Leprosy			
		**B**	**p-value**	**[95% Conf Interval]**		**B**	**p-value**	**[95% Conf Interval]**	
Physical Health	Sex	−8.838	.144	−20.832	3.156	−4.533	.548	−19.699	10.634
	Age	−.052	.776	−.42	.316	.478	.083	−.066	1.021
	Level Of Education	.83	.853	−8.21	9.871	−2.014	.762	−15.374	11.345
	Marital Status	1.569	.588	−4.256	7.395	1.431	.667	−5.248	8.11
	Annual Income (USD)	0	.169	0	0	0	.229	0	0
Psychological Health	Sex	−10.066	.226	−26.62	6.489	2.065	.678	−7.951	12.08
	Age	−.311	.222	−.818	.197	−.129	.472	−.487	.23
	Level Of Education	−7.329	.241	−19.807	5.15	.933	.831	−7.889	9.755
	Marital Status	2.682	.503	−5.359	10.722	1.48	.5	−2.93	5.891
	Annual Income (USD)	0	.962	0	0	0	.056	0	0
Social Relationships	Sex	−3.202	.635	−16.758	10.355	−4.185	.263	−11.643	3.272
	Age	−.037	.859	−.457	.383	.003	.981	−.264	.27
	Level Of Education	3.39	.504	−6.792	13.572	−2.27	.488	−8.839	4.299
	Marital Status	.119	.971	−6.54	6.779	−2.809	.091	−6.093	.475
	Annual Income (USD)	0	.603	0	0	0	p < 0.001	0	0
**0.25 Quantile**									
Physical Health	Sex	−4.168	.551	−18.197	9.86	−6.557	.063	−13.494	.38
	Age	.271	.209	−.159	.701	.184	.141	−.064	.433
	Level Of Education	−1.672	.75	−12.245	8.902	−6.338	.042*	−12.449	−.228
	Marital Status	1.339	.692	−5.474	8.153	−.814	.592	−3.869	2.24
	Annual Income (USD)	0	.617	0	0	0	p < 0.001	0	0
Psychological Health	Sex	−.297	.954	−10.747	10.153	−6.819	.239	−18.365	4.726
	Age	−.04	.803	−.36	.281	−.515	.016*	−.929	−.102
	Level Of Education	−.243	.95	−8.12	7.633	−5.531	.277	−15.701	4.639
	Marital Status	−2.691	.289	−7.766	2.385	.36	.887	−4.725	5.444
	Annual Income (USD)	0	.559	0	0	0	.051	0	0
Social Relationships	Sex	1.98	.721	−9.18	13.14	−8.701	.093	−18.922	1.519
	Age	−.136	.43	−.481	.21	−.251	.174	−.617	.116
	Level Of Education	1.19	.775	−7.193	9.573	−5.459	.227	−14.462	3.544
	Marital Status	−2.177	.426	−7.659	3.306	3.094	.172	−1.407	7.595
	Annual Income (USD)	0	.802	0	0	0	.249	0	0

*Significant at the 0.05 level (2-tailed); USD=United States Dollars.

## Data Availability

Data will be made available on request.
